# Obscure Epidemic Encephalitis

**Published:** 1919-01

**Authors:** 


**Obscure Epidemic Encephalitis.** By S. K. Vaidya. *Lancet*, Sept. 7, 1918.

Vaidya reviews the findings of eminent Americans who have worked on acute poliomyelitis, especially the phase of the blood, and cerebro-spinal fluid. He finds that the American investigators recorded a leucocytosis of from 15,000 to 30,000 in the blood, combined with an excess of protein. A well-marked excess of mononuclear cells (small lymphocytes) in the spinal fluid is always present in cases of poliomyelitis. In a small percentage of cases polynuclear cells are said to occur in the spinal fluid in the prodromal stage.

He then analyzes eighteen cases of epidemic encephalitis from the same standpoint, and comes to the following conclusions :

1. The very small amount of leucocytosis (8,000-9,000 per c. mm.) in the cases of epidemic encephalitis falls far short of the pronounced leucocytosis (15,000-30,000) recorded at the Rockefeller Institute in cases of poliomyelitis.

2. The cerebrospinal fluid is not as grossly affected as in poliomyelitis, in which often cell counts running into several hundreds and sometimes exceeding 1,000 per c. mm. have been recorded. As a matter of fact, the deviation from the normal is very small.

3. In view of the very small deviation from the normal, both of the blood and spinal fluid in cases of epidemic encephalitis, and especially in the absence of successful experimental communication of the disease to monkeys with the production of paralysis, we are not justified in asserting that the virus of epidemic encephalitis is the same as in poliomyelitis.

4. Although the results of the examinations of the blood and spinal fluid afford no positive proof in support of a diagnosis of epidemic encephalitis, such negative results will materially help in ruling out a number of obscure cerebral conditions, e. g., pneumonia, tubercular meningitis, etc., as was occasionally found to be the case.

